# Telephone Surveys Underestimate Cigarette Smoking among African-Americans

**DOI:** 10.3389/fpubh.2013.00036

**Published:** 2013-09-25

**Authors:** Hope Landrine, Irma Corral, Denise Adams Simms, Scott C. Roesch, Latrice C. Pichon, Diane Ake, Feion Villodas

**Affiliations:** ^1^Center for Health Disparities, East Carolina University, Greenville, NC, USA; ^2^Department of Psychiatric Medicine, Brody School of Medicine, East Carolina University, Greenville, NC, USA; ^3^California Black Health Network, San Diego, CA, USA; ^4^Department of Psychology, San Diego State University, San Diego, CA, USA; ^5^School of Public Health, University of Memphis, Memphis, TN, USA

**Keywords:** smoking, blacks, telephone health surveys, methodology

## Abstract

**Background:** This study tested the hypothesis that data from random digit-dial telephone surveys underestimate the prevalence of cigarette smoking among African-American adults.

**Method:** A novel, community-sampling method was used to obtain a statewide, random sample of *N* = 2118 California (CA) African-American/Black adults, surveyed door-to-door. This Black community sample was compared to the Blacks in the CA Health Interview Survey (*N* = 2315), a statewide, random digit-dial telephone survey conducted simultaneously.

**Results:** Smoking prevalence was significantly higher among community (33%) than among telephone survey (19%) Blacks, even after controlling for sample differences in demographics.

**Conclusion:** Telephone surveys underestimate smoking among African-Americans and probably underestimate other health risk behaviors as well. Alternative methods are needed to obtain accurate data on African-American health behaviors and on the magnitude of racial disparities in them.

## Introduction

Cigarette smoking contributes to cancers and other chronic diseases (1). To plan and implement strategies to reduce smoking, valid data on its prevalence are needed (1). Random digit-dial telephone surveys (RDDTS) are a common source of such data. Well-known RDDTS include the nationwide Behavioral Risk Factor Surveillance System [BRFSS (2)], the California Health Interview Survey [CHIS (3)], and state-level BRFSS (4); each is a trusted source whose data play a role in identifying health problems and allocating resources to address them. There are four methodological reasons to suspect that these and other RDDTS contain non-representative African-American/Black samples whose smoking rates underestimate Black population prevalence: (1) non-coverage bias, (2) non-response bias, (3) segregation bias, (4) and social-desirability bias.

First, a landline telephone is required to participate in RDDTS, and hence RDDTS exclude phoneless and cell-phone only households (5–8). By so doing, RDDTS inadvertently exclude many Blacks, men, young-adults, and people of low-socioeconomic status (SES) because these groups are more likely to be phoneless/cell-phone only (5–8). Moreover, people without landlines have significantly higher smoking rates. For example, among 2004–2005 and 2007 National Health Interview Survey participants [NHIS, an in-person (household) interview], smoking rates by telephone-status were phoneless = 36.9%, cell-phone-only = 32.9%, landline = 19.7% (5–8). This *non-coverage bias* raises questions about the accuracy of smoking-prevalence data from Black RDDTS samples (5–8).

Second, response rates to RDDTS are low, i.e., 10–20% (2–5). This raises the possibility of *non-response bias*, i.e., that the small percentage of people who participate differ systematically from the majority who do not (2–5). Black participation in RDDTS is even lower (9, 10). For example, a recent study of methods for recruiting Blacks to research achieved a 17.5% overall response rate (10), with 15.4% completing the mail survey, 1.9% the internet survey, and only 0.2% completing the telephone survey. Such low participation means that Black RDDTS samples are unlikely to be representative of the population. Moreover, Black RDDTS samples are 60–70% female, and hence may contain too few Black men for reliable population estimates of Black men’s smoking (2–4). Blacks’ high non-response rate (low research participation) is widely understood as a function of distrust of researchers, and dissatisfaction with their failure to improve health in the Black communities they study (11).

Third, Blacks who reside in segregated (mostly Black) neighborhoods are significantly less likely to participate in RDDTS than their non-segregated cohorts (9, 12). Because most (65–70%) of the U.S. Black population resides in segregated neighborhoods (13), their low participation raises questions about the representativeness of Black RDDTS samples (i.e., *segregation bias*) (9, 12). Moreover, segregated Blacks have significantly higher smoking rates than their non-segregated cohorts due to higher access to tobacco and tobacco-industry sponsorship of music and sporting events in Black neighborhoods (14). Segregated Blacks are similarly underrepresented in household surveys such as the NHIS (9, 12).

Finally, people tend to exhibit socially desirable responding (SDR) in household- and telephone-interview health surveys (15). SDR is the tendency to present oneself in a positive light irrespective of the veracity of that presentation (15). Common socially desirable responses in health surveys are false claims of cancer screening (16), and denial of tobacco and other substance use (17). SDR is highest in household-interviews, next highest in RDDTS, and lowest in anonymous mail surveys (15–17). SDR also is more common among Blacks (18–20). For example, in the Third National Health and Nutrition Examination Survey (NHANES-III, a household survey with biologic measures taken), Black cotinine-determined smokers were four to nine times more likely than Whites to deny smoking (20); 68% of cotinine-determined Black-women smokers denied smoking (self-reported non-smoking). Black SDR increases when actual (in-person) and presumed (internet, computer-assisted interview, RDDTS) Whites ask the questions (21). This *social-desirability bias* is a well-known threat to the validity of household and telephone health surveys of African-Americans/Blacks (18–21).

These four types of *method bias* suggest that RDDTS of Blacks acquire random but non-representative samples of mostly female, mostly older, mostly higher-SES, landline-phone owner residents of integrated neighborhoods whose smoking prevalence underestimates Black population prevalence. This implies that a new method may be needed (22, 23) to obtain large, representative samples of Black adults, and acquire the epidemiologic data that are critical to reducing health disparities. As noted, such a method must include phoneless/cell-phone-only Blacks (reduce non-coverage bias); increase Black response rates (reduce non-response bias) by decreasing distrust of researchers and giving back to the Black communities studied; include larger percentages of segregated Blacks (reduce segregation bias) and of Black men; and reduce Blacks’ high SDR (reduce social-desirability bias). Collaborating with Black community organizations and using community-based participatory research (CBPR) approaches are the recommended methods (22, 23), but have not been used to obtain random samples in epidemiologic surveys. This study is the first to use those methods to acquire a statewide, random sample of Blacks, and the first to directly compare such a sample to an RDDTS sample acquired simultaneously.

## Materials and Methods

### Participants

Participants were a random, statewide sample of *N* = 2118, US-born, self-identified Black adult residents of California (CA), 1214 women (57.3%) and 904 men (42.7%), whose ages ranged from 18 to 95 years (mean = 43.8, SD = 16.2 years).

### Procedures

Community-based sampling (CBS), CBPR approaches, and anonymous-written survey methods were used. CBS is a three-stage, random-probability, household sampling procedure used to assure inclusion of segregated, linguistically isolated, and phoneless/cell-phone only minorities, and hence more representative ethnic-minority samples (12, 24, 25). In Stage 1, census data were used to identify the counties in which the majority of CA Blacks reside. This revealed that most (94%) of the CA Black population resided in seven counties, e.g., Los Angeles (42%), Riverside (10%), Sacramento (10%). Blacks were sampled proportional to county representation: 42% of the sample was obtained in Los Angeles county and 10% in Riverside county (etc.), such that the sample matched the CA Black population. This was achieved by sampling more or fewer census tracts in each county as needed (25).

In Stage 2, 513 census tracts (CTS) within the 7 counties were randomly selected. In Stage 3, equal numbers of low- (20–50% Blacks) and high-segregated (60–92% Blacks) CTS were randomly selected from the 513, and block-groups within those then randomly selected. Every household in the block-groups was sampled door-to-door on weekends, with one adult participant permitted per household. The door-to-door method assured inclusion of the phoneless/cell-phone-only. Further details on the method are provided elsewhere (25).

The CBPR aspect of the study was collaboration with the California Black Health Network (CBHN), a well-known, trusted, community organization that has conducted health promotion programs statewide for CA Blacks since the 1970s. CBHN needed a statewide health-assessment to improve its programs, and so co-sponsored the study. CBHN hired Black surveyors from each community to collect data in that community. Surveyors wore CBHN ID badges, approached all households in the block-groups, introduced themselves as CBHN staff, and stated that the purpose of the survey was to acquire data needed to improve CBHN programs in each Black community. Surveyors handed potential participants an Informed Consent Letter that described the survey, stated this study purpose, and included CBHN phone numbers (in each county) to call. Surveyors then asked if a Black adult resided in the household who might wish to complete the anonymous, CBHN health survey for $10 cash. Using these CBPR approaches, the response rate was 99%, i.e., of those who answered the door, 99% completed and only 1% refused the survey (25).

To further reduce social-desirability bias, the novel approach of distributing an anonymous written survey (similar to mail-surveys) was used instead of interviews. Surveys were left with participants to complete in private, and retrieved 20–30 min later. The study had the approval of the Institutional Review Board of San Diego State University.

### Measures

The survey asked standard (NHIS, BRFSS) questions about health behavior (diet, exercise) and about tobacco use: have you smoked 100 cigarettes or more in your entire life (yes/no), and do you smoke cigarettes now, even once in a while (yes/no). Telephone status (phoneless, cell-phone-only, landline) and demographic variables also were assessed.

### Data analyses

Analyses compare the 2007 community-based (CBS) Black sample (*N* = 2118) to the Black (*N* = 2315) and White (*N* = 31,388) samples in the 2007 CA Health Interview Survey [CHIS (3)]. The CHIS was a statewide, RDDTS of *N* = 51,048 CA adults conducted simultaneously (2006–2007). Chi-square and ANOVAs were used to compare samples, with follow-up *z-* and *t-*tests used for pairwise comparisons of proportions and means, respectively.

## Results

Table 1 compares the CBS and RDDTS Black samples on demographics and cigarette smoking. As shown, the CBS sample was more diverse in age, and was younger; 58.4% of CBS versus 77% of RDDTS Blacks were ≥age 40. The CBS sample also contained significantly larger percentages of Black men and of low-income adults. Smoking prevalence among CBS Blacks was 32.6%, and significantly higher than that of RDDTS Blacks (19.1%), for women (CBS = 29.7% vs. RDDTS = 18%) and men (CBS = 37.2% vs. RDDTS = 21.1%).

**Table 1:** **Demographics and smoking among community (CBS) and telephone (RDDTS) Black samples**.

	CBS Blacks (*N* = 2118)	RDDTS Blacks (*N* = 2315)	Significance test	*p*
**Age**
Mean (SD)	43.8 (16.4)	52.3 (17.0)	*F*_1,4104_ = 264.44	<0.001
Range	18–95	18–85		
**Age groups**			$\chi_{\text{df = 2}}^{2}=169.00$	<0.001
18–25 years	16.8%	7.8%	*z* = 8.57	<0.001
26–39 years	24.8%	15.2%	*z* = 7.66	<0.001
40 years and older	58.4%	77.0%	*z* = 12.75	<0.001
**Gender**			$\chi_{\text{df = 1}}^{2}=29.96$	<0.001
Men	42.7%	34.8%	*z* = 5.16	<0.001
Women	57.3%	65.2%	*z* = 5.16	<0.001
**Income**			$\chi_{\text{df = 3}}^{2}=55.68$	<0.001
$10,999 and lower	21.7%	13.2%	*z* = 7.07	<0.001
$11,000–25,999	18.4%	21.5%	*z* = 2.33	0.02
$26,000–49,999	23.6%	23.0%	*z* = 0.37	0.71
$50,000 and higher	36.3%	42.3%	*z* = 3.82	<0.001
**Education**			$\chi_{\text{df = 2}}^{2}=9.95$	0.007
Less than high school (HS)	6.8%	9.2%	*z* = 2.79	0.005
HS Grad or GED	28.2%	25.6%	*z* = 1.79	0.07
College and higher	65.1%	65.1%	*z* = 0.03	0.98
**Current smoking**
Overall % yes	32.6%	19.1%	$\chi_{\text{df = 1}}^{2}=97.98$	<0.001
Men	37.2%	21.1%	*z* = 6.77	<0.001
Women	29.7%	18.0%	*z* = 6.73	<0.001

About 83% of the CBS sample answered questions on their telephone status (*n* = 1752). Of these, 13% (*n* = 230) were phoneless or cell-phone only (P/CPO). Table 2 compares CBS P/CPO to CBS-landline and RDDTS Blacks. As shown, P/CPO Blacks (mean age = 37.7) were significantly younger than CBS-landline (mean age = 45.3) and RDDTS Blacks (mean age = 52.3). The P/CPO Black sample also contained a significantly larger percentage of men (56%) than the CBS-landline (40.6%) and RDDTS (34.8%) samples, and had significantly lower incomes: 45.2% of P/CPO Blacks were in the lowest-income group vs. 13.2% of RDDTS Blacks. Smoking prevalence among P/CPO Blacks was 50.2 vs. 29.5% for CBS-landline and 19.1% for RDDTS Blacks.

**Table 2:** **Demographics and smoking among CBS vs. RDDTS Black samples by telephone status**.

	Group 1 CBS P/CPO (*N* = 230)	Group 2 CBS landline (*N* = 1522)	Group 3 RDDTS Blacks (*N* = 2315)	Significance test	*p*
**Age**
Mean (SD)	37.7 (13.9)	45.3 (16.3)	52.3 (17.0)	*F*_2,3941_ = 132.07	<0.001
				*t*^Groups 1,2^ = 7.15	<0.001
				*t*^Groups 1,3^ = 14.31	<0.001
				*t*^Groups 2,3^ = 12.57	<0.001
**Age groups**				$\chi_{\text{df = 4}}^{2}=175.50$	<0.001
18–25 years	24.5%	14.1%	7.8%	*z*^Groups 1,2^ = 3.81	<0.001
				*z*^Groups 1,3^ = 7.96	<0.001
				*z*^Groups 2,3^ = 6.15	<0.001
26–39 years	32.5%	23.8%	15.2%	*z*^Groups 1,2^ = 2.67	0.008
				*z*^Groups 1,3^ = 6.39	<0.001
				*z*^Groups 2,3^ = 6.51	<0.001
40 years and older	42.9%	62.1%	77.0%	*z*^Groups 1,2^ = 5.23	<0.001
				*z*^Groups 1,3^ = 10.77	<0.001
				*z*^Groups 2,3^ = 9.75	<0.001
**Gender**				$\chi_{\text{df = 2}}^{2}=44.03$	<0.001
Men	56.0%	40.6%	34.8%	*z*^Groups 1,2^ = 4.21	<0.001
				*z*^Groups 1,3^ = 6.13	<0.001
				*z*^Groups 2,3^ = 3.15	<0.001
Women	44.0%	59.4%	65.2%	*z*^Groups 1,2^ = 4.21	<0.001
				*z*^Groups 1,3^ = 6.13	<0.001
				*z*^Groups 2,3^ = 3.55	<0.001
**Income**				$\chi_{\text{df = 6}}^{2}=153.60$	<0.001
Less than $10,999	45.2%	17.1%	13.2%	*z*^Groups 1,2^ = 9.18	<0.001
				*z*^Groups 1,3^ = 11.99	<0.001
				*z*^Groups 2,3^ = 3.17	0.002
$11,000–25,999	16.8%	18.8%	21.5%	*z*^Groups 1,2^ = 0.57	0.57
				*z*^Groups 1,3^ = 1.48	0.14
				*z*^Groups 2,3^ = 1.93	0.05
$26,000–49,999	18.3%	24.8%	23.0%	*z*^Groups 1,2^ = 1.98	0.05
				*z*^Groups 1,3^ = 1.48	0.14
				*z*^Groups 2,3^ = 1.20	0.23
$50,000 and higher	19.7%	39.3%	42.3%	*z*^Groups 1,2^ = 5.34	<0.001
				*z*^Groups 1,3^ = 6.28	<0.001
				*z*^Groups 2,3^ = 1.75	0.08
**Education**				$\chi_{\text{df = 4}}^{2}=41.88$	<0.001
Less than high school	11.1%	6.1%	9.2%	*z*^Groups 1,2^ = 2.60	0.009
				*z*^Groups 1,3^ = 0.80	<0.001
				*z*^Groups 2,3^ = 3.31	<0.001
HS Grad or GED	40.7%	26.2%	25.6%	*z*^Groups 1,2^ = 4.46	<0.001
				*z*^Groups 1,3^ = 4.78	<0.001
				*z*^Groups 2,3^ = 0.31	0.76
College and higher	48.2%	67.7%	65.1%	*z*^Groups 1,2^ = 5.65	<0.001
				*z*^Groups 1,3^ = 4.97	<0.001
				*z*^Groups 2,3^ = 1.57	0.12
**Current smoking**
Overall%	50.2%	29.5%	19.1%	$\chi_{\text{df = 2}}^{2}=129.70$	<0.001
				*z*^Groups 1,2^ = 5.89	<0.001
				*z*^Groups 1,3^ = 10.38	<0.001
				*z*^Groups 2,3^ = 7.25	<0.001
**Current smoking**
Men	47.7%	32.9%	21.1%	*z*^Groups 1,2^ = 2.42	0.02
				*z*^Groups 1,3^ = 5.90	<0.001
				*z*^Groups 2,3^ = 5.54	<0.001
Women	53.4%	25.3%	18.0%	*z*^Groups 1,2^ = 5.18	<0.001
				*z*^Groups 1,3^ = 7.94	<0.001
				*z*^Groups 2,3^ = 4.62	<0.001

The differences (Table 1) between the CBS and RDDTS samples on demographic and telephone variables might account for their differences in smoking rates. To examine this, a hierarchical logistic regression predicting smoking among the combined samples was conducted, with demographic and telephone variables entered on Step 1, and Sample on Step 2. As shown in Table 3, demographic and telephone variables contributed to smoking. The odds of smoking were higher for those with low educations and incomes, ages 26–39, men, and the P/CPO. Adding Sample on Step 2 significantly improved model fit ($\chi_{\text{df = 1}}^{2}=52.89,p\text{ < 0.001}$), and revealed that the odds of smoking were higher for CBS than RDDTS Blacks: Even after controlling for demographic and telephone differences between the samples, CBS Blacks remained nearly twice as likely to be smokers than RDDTS Blacks.

**Table 3:** **Logistic regression predicting smoking among CBS and RDDTS Blacks**.

Predictor and step	β	*p*	OR	95% CI
**STEP 1**				
Education (reference = college and more)
HS Grad/GED	0.52	<0.001	1.67	1.39, 2.01
Less than HS	0.44	<0.001	1.55	1.16, 2.07
Income (reference = $50,000 and higher)
$26,000–49,999	0.42	<0.001	1.52	1.22, 1.90
$11,000–25,999	0.82	<0.001	2.26	1.81, 2.83
$10,999 and lower	0.88	<0.001	2.41	1.88, 3.10
Age (reference = 40 years and older)
26–39 years	0.24	0.02	1.27	1.03, 1.55
18–25 years	−0.29	0.04	0.75	0.58, 0.98
Gender (reference = women)
Men	0.37	<0.001	1.45	1.23, 1.71
Telephone (reference = landline)
Phoneless and cell-phone-only	1.00	<0.001	2.72	1.94, 3.83
**STEP 2**				
Sample (reference = RDDTS)
CBS	0.65	<0.001	1.91	1.61, 2.28

Table 4 compares smoking prevalence among CBS Blacks vs. RDDTS Blacks and Whites. As shown, the smoking rate among RDDTS Blacks (19.1%) was significantly higher than that of RDDTS Whites (13%), but the difference was small, i.e., 6% points. Smoking prevalence among CBS Blacks (32.6%) also was significantly higher than that of RDDTS Whites, but the difference was large, i.e., 19.5% points.

**Table 4:** **Smoking among CBS blacks vs. RDDTS Blacks and Whites**.

Current smoking	Group 1 CBS Blacks (*N* = 2,118) (%)	Group 2 RDDTS Blacks (*N* = 2,315) (%)	Group 3 RDDTS Whites (*N* = 31,388) (%)	Significance test	*p*
Overall % Yes	32.6	19.1	13.1	$\chi_{\text{df = 2}}^{2}=557.2$	<0.001
				*z*^Groups 1,2^ = 9.75	<0.001
				*z*^Groups 1,3^ = 22.67	<0.001
				*z*^Groups 2,3^ = 8.10	<0.001
Men	37.2	21.1	14.5	*z*^Groups 1,2^ = 6.88	<0.001
				*z*^Groups 1,3^ = 16.29	<0.001
				*z*^Groups 2,3^ = 5.09	<0.001
Women	29.7	18.0	12.2	*z*^Groups 1,2^ = 6.73	<0.001
				*z*^Groups 1,3^ = 15.64	<0.001
				*z*^Groups 2,3^ = 6.52	<0.001

## Discussion

This study has four important results. First, an unprecedented 99% participation rate among African-American/Black adults was achieved. Factors that probably contributed to this were CBHN sponsorship of the study; use of an anonymous, written survey, and of Black surveyors from the communities studied; and CBHN’s commitment to return to improve health in those communities. Participants’ comments revealed that the latter was the key: again and again, participants said that they were weary of being “studied,” wanted something to be done, and would participate because CBHN intended to do something for them. That about 15% of participants refused the $10 incentive and instructed us to use it for CBHN health programs is consistent with participant comments. If this explains the high participation rate, then it suggests that one effective way to increase Blacks’ participation in research might be to *give something to the communities studied*.

Because the response rate was high, over-sampling was not needed; census tracts were sampled once. Consequently, the cost of the study was about $100 per participant (incentive included). This is far less than RDDTS [about $400 per participant in the CHIS3 (3), with no monetary incentive] in which Black households must be telephoned repeatedly to acquire a similar sample size (*N* = 2118 here, *N* = 2315 in the CHIS sample).

A second notable finding is that 13% of the sample reported being phoneless/cell-phone only (P/CPO). Although substantial, only 83% of participants answered the telephone-status question. If those who didn’t answer also were P/CPO, then the P/CPO rate among CA Blacks might have been as high as 30%. In any event, smoking rates among P/CPO Blacks – those who cannot participate in RDDTS – were extremely high, 53% among women, 48% among men. This raises serious doubts about the accuracy of RDDTS data on Black smoking prevalence and the nature of gender differences in it.

The third notable finding is that the CBS sample was more representative of the CA Black population (more similar to census data (26)) than the RDDTS sample. As expected, the RDDTS Black sample consisted of mostly older (77% >age 40) women (65%), whereas the CBS sample contained significantly more young adults and Black men. Indeed, men comprise 48.7% of the CA Black population (26), and 42.7% of the CBS sample, but only 34.8% of the RDDTS sample.

Finally, the fourth important finding was that smoking prevalence among CBS Blacks (32.6%) was significantly higher than that of the RDDTS Blacks (19.1%) sampled simultaneously, and remained twice as high after controlling for differences between the samples in income, education, gender, age, and telephone-status. Smoking-prevalence differences were not an artifact of those factors, and hence are probably a function of survey method. This in turn suggests that the RDDTS method significantly underestimates smoking among Blacks, and by implication, may underestimate other health risk behaviors as well. This interpretation is consistent with growing concerns about using RDDTS with low-SES and Black populations (5–9). Thus, we urge caution in using RDDTS data to draw conclusions about smoking (and other health risk behaviors) among Blacks.

Why were smoking rates significantly higher among CBS Blacks even after controlling for sample demographics and telephone-status? One possibility is that segregated Blacks were deliberately included in the CBS sample but not in the RDDTS sample. Smoking rates are significantly higher among segregated Blacks due to higher access to tobacco, greater exposure to targeted tobacco-advertising, and tobacco-industry sponsorship of events in segregated neighborhoods (14, 27). However, multi-level modeling (not shown) found no association between segregation and smoking within the CBS sample. Nonetheless, it is possible that the segregation level of CBS Blacks was higher than that of RDDTS Blacks and contributed to higher smoking among the former. If this is the case, then it highlights the need to include more segregated Blacks in epidemiologic surveys (9, 12), and underscores the non-representativeness of RDDTS Black samples.

An alternative, perhaps more likely explanation for the findings is that there was significantly lower SDR – i.e., denial of smoking – among CBS than among RDDTS Black smokers, for two reasons. First, an anonymous, written survey rather than a telephone or in-person interview survey was used. Ample data indicate that the latter methods elicit higher SDR and higher denial of smoking, among Blacks in particular (15, 17, 18, 20). Second, our surveyors were Blacks, and studies indicate that they elicit lower SDR from Black respondents (18, 21). If low SDR among CBS Blacks explains their higher smoking rate, then it suggests that *anonymous, written surveys distributed to Blacks by Blacks* may increase the validity of Blacks’ self-reported smoking.

The latter point highlights a problem inherent in all surveys of health behavior, namely, their reliance on self-reports that are subject to SDR, and the possibility that disparities-data based on such reports are attenuated by Blacks’ differentially high SDR. In the larger social context of racial stratification, segregation and discrimination, many Blacks (justifiably) distrust White researchers (11), and so may provide self-protective, socially desirable responses that (ironically) obscure their health behaviors and health needs. This implies that the prevalence of smoking and other health risk behaviors among Black adults, and the magnitude of Black-White health-behavior disparities may be unknown. Moreover, given that SDR is higher in household- than in telephone-interviews (15, 20), we also urge caution in using self-report data from household surveys (e.g., NHANES-III, NHIS) to draw conclusions about the prevalence of smoking and other health behaviors among Blacks (20).

The 33% smoking-prevalence rate found also has implications for understanding Black-White cancer disparities. For the past 25 years, RDDTS and household surveys alike have found that smoking rates are 2–6% points higher among Blacks than Whites (28), a finding replicated here when comparing RDDTS Blacks and Whites (Table 4). This finding is troublesome because the difference is too small to account for large Black-White disparities in smoking-related cancers and diseases, and too small to indicate a need for targeted tobacco-cessation programs for Blacks (28). The data here suggest that the Black-White smoking-prevalence difference might be as large as 19% points, a difference that (if consistent for prior generations) might explain racial disparities in incidence of smoking-related diseases. In any event, the 33% smoking-prevalence rate underscores the need for smoking-cessation programs for CA Blacks, and for increased funding opportunities for those.

These interpretations must be considered in the context of the limitations of this study. One obvious limitation is that we did not measure SDR, and hence only can speculate that it contributed to sample differences in self-reported smoking. Replications that assess SDR are needed to clarify this, but must use SDR scales with caution in light of their lack of cross-cultural measurement equivalence (18). A similar limitation is the lack of segregation data for the RDDTS sample, these unavailable in the CHIS. Hence we only can speculate that possible sample differences in segregation might have contributed to differences in smoking. Likewise, we speculated but did not demonstrate that the differences in smoking for the CBS vs. RDDTS samples generalize to other health risk behaviors among Blacks as well. However, given that the four types of method bias inherent in RDDTS of Blacks remain irrespective of the specific health behavior examined, this speculation is both logical and reasonable. In addition, recent RDDTS health surveys have begun to include cell-phone-only households. This methodological improvement however does not resolve the other three method problems inherent in RDDTS of Blacks. Finally, smoking rates may have changed between this study and the present.

Despite these limitations, the study has several strengths. These include a random, representative, statewide sample of >2,000 Black adults; development of a novel, inexpensive, efficient, health-survey methodology that has the potential to advance research on Blacks; and the first direct demonstration of the troubles inherent in using the RDDTS method with Blacks. Thus, we encourage replication of this study with multiple, self-reported health behaviors and chronic diseases. Such replications have the potential to improve the epidemiologic data on health and health disparities that are critical to developing targeted interventions, and essential to demonstrating the need for them.

## Author’s Contribution

Hope Landrine, PhD: (1) conception and design of study; supervision of data collection; data analyses and interpretation; (2) drafting the manuscript, critical revision of the manuscript; (3) final approval of the submitted version. Irma Corral, PhD, MPH: (1) conception and design of study; supervision of data collection; data analyses and interpretation; (2) drafting the manuscript, critical revision of the manuscript; (3) final approval of the submitted version. Denise Adams Simms, MPH: (1) conception and design of study; supervision of data collection; data analyses and interpretation; (2) drafting the manuscript, critical revision of the manuscript; (3) final approval of the submitted version. Scott Roesch, PhD: (1) data analyses and interpretation; supervision of data collection; (2) drafting the manuscript, critical revision of the manuscript; (3) final approval of the submitted version. Latrice C. Pichon, Ph.D., MPH: (1) data analyses and interpretation; data collection; (2) drafting the manuscript, critical revision of the manuscript; (3) final approval of the submitted version. Diane Ake, MPH: MPH: (1) data analyses and interpretation; data collection; (2) drafting the manuscript, critical revision of the manuscript; (3) final approval of the submitted version. Feion Villodas, MS, MPH: MPH: (1) data analyses and interpretation; data collection; (2) drafting the manuscript, critical revision of the manuscript; (3) final approval of the submitted version.

## Conflict of Interest Statement

The authors declare that the research was conducted in the absence of any commercial or financial relationships that could be construed as a potential conflict of interest.
